# Gathering the Stakeholder’s Perspective: Experiences and Opportunities in Rare Genetic Disease Research

**DOI:** 10.3390/genes14010169

**Published:** 2023-01-07

**Authors:** Lauren K. White, T. Blaine Crowley, Brenda Finucane, Emily J. McClellan, Sarah Donoghue, Sixto Garcia-Minaur, Gabriela M. Repetto, Matthias Fischer, Sebastien Jacquemont, Raquel E. Gur, Anne M. Maillard, Kirsten A. Donald, Anne S. Bassett, Ann Swillen, Donna M. McDonald-McGinn

**Affiliations:** 1 Children’s Hospital of Philadelphia, Philadelphia, PA 19104, USA; 2 Perelman School of Medicine, University of Pennsylvania, Philadelphia, PA 19104, USA; 3 Geisinger Medical Center, Danville, PA 17821, USA; 4 Institute of Medical and Molecular Genetics (INGEMM), La Paz University Hospital, 28046 Madrid, Spain; 5 Clinic Aldemana, University for Development, Santiago 7690000, Chile; 6 Clinic and Policlinic for Psychiatry and Psychotherapy, University of Rostock, 18147 Rostock, Germany; 7 Sigma-Zentrum, 79713 Bad Säckingen, Germany; 8 Sainte Justine Research Center, University of Montreal, Montreal, QC H3T 1J4, Canada; 9 Lausanne University Hospital, 1011 Lausanne, Switzerland; 10 Department of Paediatrics and Child Health, Red Cross War Memorial Children’s Hospital, Rondebosch, Cape Town 7700, South Africa; 11 Neuroscience Institute, University of Cape Town, Cape Town 7935, South Africa; 12 The Dalglish Family 22q Clinic, University Health Network, Toronto, ON M5G 2C4, Canada; 13 Clinical Genetics Research Program and Campbell Family Mental Health Research Institute, Centre for Addiction and Mental Health, and Department of Psychiatry, University of Toronto, Toronto, ON M5S 2S1, Canada; 14 Division of Cardiology, Department of Medicine, and Centre for Mental Health, and Toronto General Hospital Research Institute, University Health Network, Toronto, ON M5G 2N2, Canada; 15 Center for Human Genetics, University Hospital UZ Leuven, and Department of Human Genetics, KU Leuven, 3000 Leuven, Belgium; 16 Department of Human Biology and Medical Genetics, Sapienza University, 00185 Roma, Italy

**Keywords:** copy number variations (CNVs), neurodevelopmental psychiatric disorders (NPDs), 22q11.2 deletion syndrome

## Abstract

Background: Research participant feedback is rarely collected; therefore, investigators have limited understanding regarding stakeholders’ (affected individuals/caregivers) motivation to participate. Members of the Genes to Mental Health Network (G2MH) surveyed stakeholders affected by copy number variants (CNVs) regarding perceived incentives for study participation, opinions concerning research priorities, and the necessity for future funding. Respondents were also asked about feelings of preparedness, research burden, and satisfaction with research study participation. Methods: Modified validated surveys were used to assess stakeholders´ views across three domains: (1) Research Study Enrollment, Retainment, Withdrawal, and Future Participation; (2) Overall Research Experience, Burden, and Preparedness; (3) Research Priorities and Obstacles. Top box score analyses were performed. Results: A total of 704 stakeholders´ responded from 29 countries representing 55 CNVs. The top reasons for initial participation in the research included reasons related to education and altruism. The top reasons for leaving a research study included treatment risks and side effects. The importance of sharing research findings and laboratory results with stakeholders was underscored by participants. Most stakeholders reported positive research experiences. Conclusions: This study provides important insight into how individuals and families affected with a rare CNV feel toward research participation and their overall experience in rare disease research. There are clear targets for areas of improvement for study teams, although many stakeholders reported positive research experiences. Key findings from this international survey may help advance collaborative research and improve the experience of participants, investigators, and other stakeholders moving forward.

## 1. Introduction

Participant feedback from research subjects is rarely collected. Thus, investigators have limited data on the motivation, satisfaction, and overall perspectives of research participants. Samples sizes are often small and there exist few validated tools available to produce actionable insights for investigative teams [1]. Factors contributing to the recruitment and retention of research participants remain relatively unknown [2,3]. In contrast, in clinical care, patient-centric outcome metrics are well-established indicators for improved care and outcomes, as well as patient satisfaction [4]. As clinical care has used such metrics to improve patient satisfaction, it is imperative that research programs implement procedures that contribute to positive and meaningful experiences for participants. This information will also benefit future research strategies and study designs to increase participation and improve outcomes of both patient families and research teams [5,6]. Such efforts are especially pertinent for rare disease populations, which include individuals affected by chromosomal deletions or duplications (pathogenic copy number variants; CNV) [7,8,9,10,11].

Although generalized tools and baseline datasets examining research participants’ experiences have become more available over the last decade e.g., [12], significant gaps remain. First, these studies primarily focus on research being conducted in the United States and Europe. Second, this work has not specifically targeted participants with rare genetic diseases. Understanding research motivation, obstacles, and experiences of stakeholders affected by rare CNVs from an international perspective are important for several reasons. First, the population base rate for many rare CNVs is below 1%. For instance, the most common rare CNV is 22q11.2 deletion syndrome, estimated to occur in 1:2148 live births [13]. Thus, many research studies have low sample sizes; understanding participants’ experiences and improving their research satisfaction may help increase sample size and improve scientific findings within CNV populations. Additionally, individuals with rare CNVs often have many comorbidities resulting in increased cognitive, psychological, and medical burden [10,14,15,16]. Understanding stakeholders’ research experiences may help reduce the burden added by investigative teams. 

Lead investigators from the Genes to Mental Health Network (G2MH), an initiative funded by the United States of America’s National Institute of Mental Health and the Eunice Kennedy Shriver National Institute of Child Health and Human Development encompassing researchers from 14 institutions and seven countries across North America, Europe, and Africa, aimed to estimate the level of participation and overall experience in rare disease research, gather data on factors that motivate families to join, leave, and remain in research studies, and collect participant and caregivers´ opinions on research priorities and obstacles to participation. Lastly, the investigators sought to understand whether previous participants felt valued as a part of the study process. 

## 2. Materials and Methods

### 2.1. Procedures

The study was led by G2MH principal investigators (BF, KD, ASB, AS, and DMM) who formed a “Stakeholders Committee” in April 2020 with international collaborators participating from 10 institutions across three continents. The committee began by reviewing available validated tools to be used in the study and selected The Research Participant Perception Survey [12] and the Rare Barometer Survey [17] for use in the current study. All items were originally programmed in English. Translations to five languages were generated utilizing the DeepL AI translator and verified by a native speaker resulting in surveys in: English, Dutch, French, German, and Spanish (both European and South American dialects separately). Each version of the instruments was built and administered in REDCap by TCB. All data was managed and stored on servers at the Children’s Hospital of Philadelphia (CHOP). IRB review was deemed exempt by the CHOP IRB. Consent to participate was acknowledged by each participant when entering the survey. 

**Figure 1:**
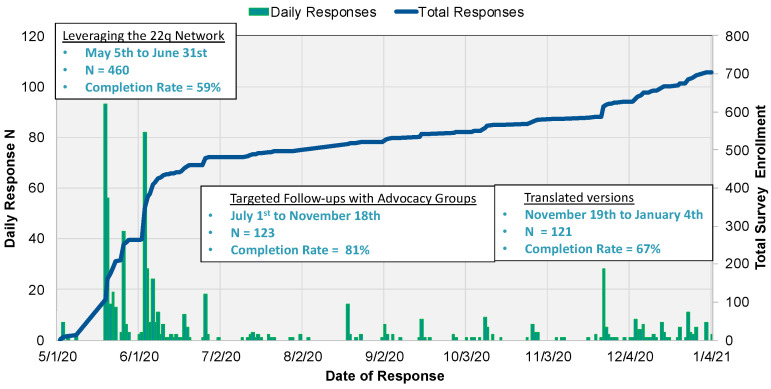
Study Enrollment Over Time.

Distribution of the survey occurred in three distinct phases (see Figure 1) beginning in May 2020 and concluding in January 2021. Phase I (May–June 2020) leveraged the existing network of chromosome 22q11.2 Deletion Syndrome collaborators, as 7 of the 10 lead investigators were members of the International 22q11.2 Brain and Behavior Consortium (IBBC) and 22q11.2 Society, and family/advocate organizations, while Phase II (July 2020–October 2020) allowed for targeted follow-ups with other rare CNV organizations without an existing, established relationship. Phase III (November 2020–January 2021) deployed the 5 non-English versions of the survey. 

### 2.2. Participants

**Figure 2:**
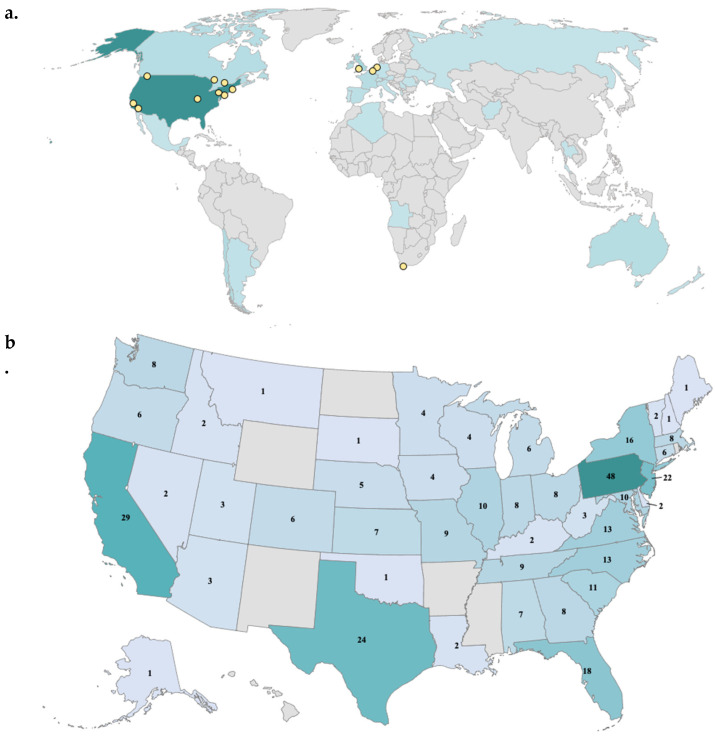
Global Stakeholder Survey Responses and Genes to Mental Health Network Locations. **Notes**. Panel (**a**) shows responses by country. A total of 29 unique countries were represented in the survey responses. Over half of all survey responses originated from the United States (N = 358, 51%). The next eight highest-responding countries combined to account for over a quarter (28%) of the total sample: United Kingdom (N = 54), Chile (N = 35), Australia (N = 25), Canada (N = 24), Belgium (N = 24), France (N = 23), Ireland (N = 21), and Spain (N = 14). In total, 12% of all respondents declined to answer. Panel (**b**) shows responses within the United States. Responses were recorded in 44 states, with a slight concentration in the Mid-Atlantic and Northeast regions. The five states with member institutions in the G2MH Network accounted for 29% of the US sample: Pennsylvania (N = 48), California (N = 29), Missouri (N = 9), Washington, and Massachusetts (both N = 8).

A total of 704 surveys (68% of all initiated) were completed by participants from 29 countries. The majority of participants identified as an unaffected family member (82% parent; 5% as another family member). Affected individuals made up 9% of study participants, with 4% identifying as an individual with a CNV and 5% identifying as an affected parent. A total of 85% of participants were female, the majority being mothers of a child with a CNV. The demographic distribution of responders approximated that of the sample population of the consortium, with additional countries represented in a small subset (Figure 2a). Responses gathered from the United States were distributed across many states, though mostly concentrated in the Northeast (Figure 2b). See Table 1 and Table 2 for participant demographics and CNVs of the sample.

### 2.3. Research Participation Survey

The research instrument employed items from two established surveys: The Research Participant Perception Survey [12] and the Rare Barometer Survey [17]. The resulting survey included items across three domains: (1) Research Study Enrollment, Retainment, Withdrawal, and Future Participation; (2) Overall Research Experience, Burden, and Preparedness; (3) Research Priorities and Obstacles. 

For the Section 3.1, participants were presented with reasons for joining a research study (13 items), staying/continuing in a study (*n* = 16 items), and withdrawing from a study (14 items). They were asked to rate each item from 1 (not important) to 4 (very important). The staying and leaving items were only administered to individuals who endorsed previously participating in a research study. Participants were also asked to select which items from a list of ten items were important when considering future study participation. 

For the items within the Section 3.2, participants were asked to choose their overall experiences from 0 (worst possible experience) to 10 (best possible experience). Participants were also asked to endorse the level of burden using simple, moderate, intense responses as well as their preparedness for research by the study team from 1 (no, not prepared) to 4 (yes, completely prepared). Participants were asked to rate if they felt similar to a valued research partner from 1 (never) to 4 (always) and if they would recommend research participation to friends and family from 1 (no) to 4 (definitely, yes). 

For items within the Section 3.3, participants were provided with a list (*n* = 7) of items and asked to rate each item from 1 (lowest research priority) to 10 (highest research priority). For perceived obstacles, participants were asked to rank the top three highest perceived obstacles for conducting research from a list of eight items. 

### 2.4. Data Analysis

All survey items were scored using the “Top-Box” method using procedures outlined in Kost et. al 2014. The “top-box” method reports the most favorable/optimal (highest) response for a given item. The total percentage of participants who selected the “Top-Box” response is reported. Moreover, the data analyzed in the “Top-Box” were all “positive” responses (Kost. 2014), where no-response, “prefer not to say” and “I don’t know” responses were removed from analyses. In general, questions asking for a singular 1–10 ranking analyses used the top two responses as “Top-Box” (combining ratings of 9 and 10), while the items with a 1–4 or smaller range of responses only used the highest/most optimal response as the “Top-Box”. For items that did not have optimal responses (e.g., perceived burdens to research) we report the most endorsed items. On the survey, responses across items were not required (i.e., mandatory), so the missing data per item was not evenly distributed. For each statistic, the percentage of positive responses (removing ‘prefer not to say’, ‘don’t know’, and skipped responses) as well as Ns are reported. Differences in survey responses by region are presented in Supplemental Materials.

## 3. Results

### 3.1. Research Study Enrollment, Retainment, Withdrawal, and Future Participation 

**Figure 3:**
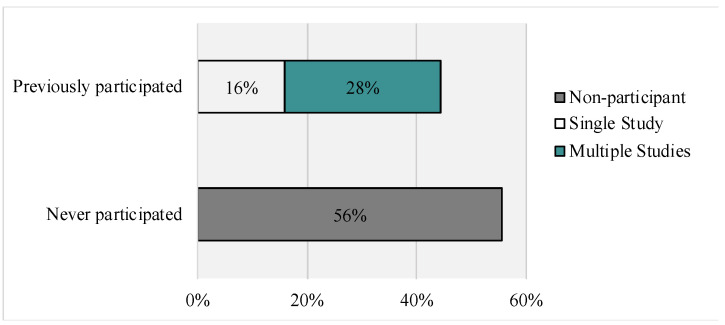
Prior Research Participation.

**Figure 4:**
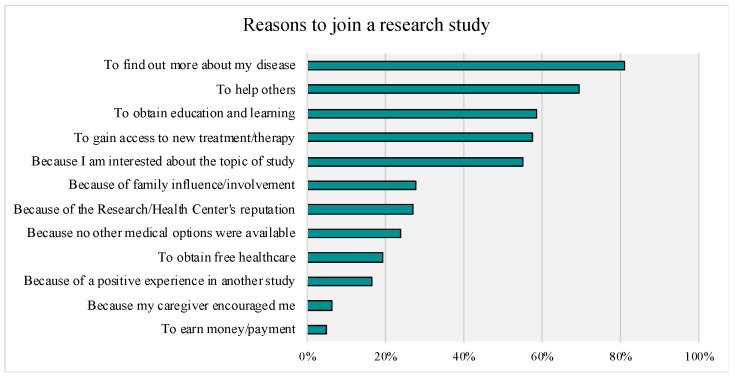
Rankings of Reasons for Joining a Research Study. Top Box Responses (“very important”) are Presented.

**Figure 5:**
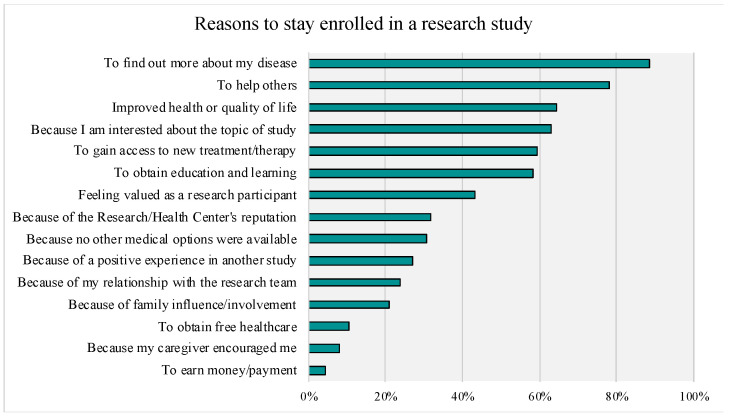
Rankings of Reasons for Staying Enrolled in a Research Study. Top Box Responses (“Very Important”) are Presented.

Of those respondents who reported their prior research participation (see Figure 3), the majority (56%, n = 345) reported never participating in research; 16% (n = 98) reported participating in one prior study; 28% (n = 136) reported participating in two or more studies. For those that reported on types of prior research participation, the following were reported: research to develop treatment/therapies (clinical trials research): 11.1 % (n = 78); research on the quality of life: 15.2 % (n = 107); research to develop genetic therapies: 5.5% (n = 39); research to develop medical devices: 1.6% (n = 11); market research: 1.0% (n = 7); other: 8.2% (n = 58). For reasons to join a study, the item most often marked as “very important” (Top-Box) was “to find out more about my disease”, followed by “to help others”. Access to new treatments and therapy, interest in the topic of research, and learning/obtaining education were also rated as “very important” by over 50% of respondents. Top-Box percentages for the given reasons to enroll and continue in research are presented in Figure 4 and Figure 5, respectively. 

**Figure 6:**
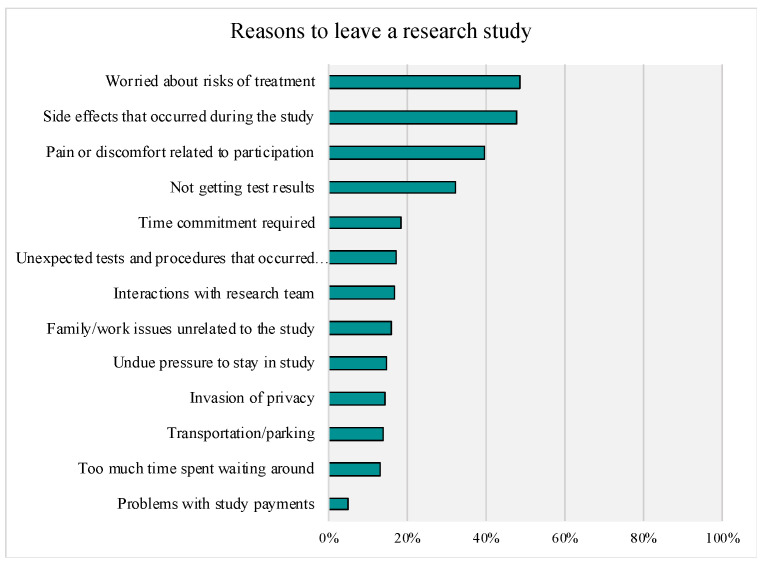
Reasons for Withdrawing from a Research Study. Top Box Responses (“Very Important”) are Presented.

When participants were asked to report on reasons why they would withdrawal from a study, ‘Risks of treatment’ and ‘Study side effects’ were the most common responses with items receiving 49% and 48% of top-box responses, respectively. Top-Box percentages for reasons to withdraw are presented in Figure 6. Of note, the overall percentage of participants selecting the top-box (“very important”) response was low across study withdrawal items. 

**Figure 7:**
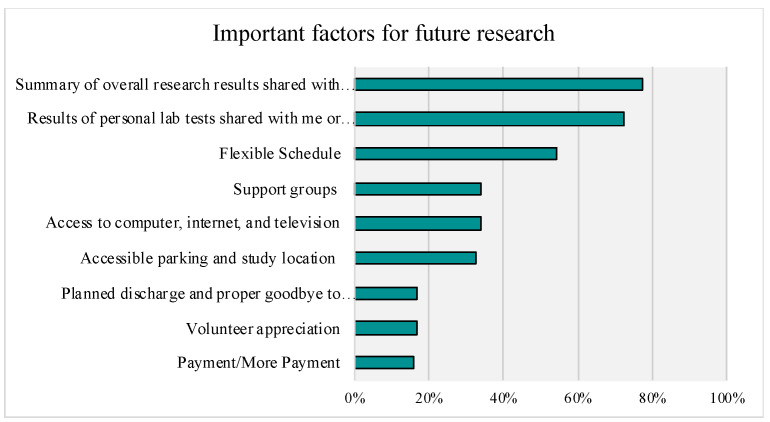
Important Factors for Future Research Participation. Top-Box Answers are Presented (Rankings of 9 and 10).

Of note, in terms of areas that were ranked as important for consideration in future research participation, the two most valued factors were if the research (77%) or lab (72%) results were to be shared with the participant or their healthcare provider (see Figure 7 for the full list of top-box endorsements). 

### 3.2. Overall Research Experience, Burden, and Preparedness 

**Figure 8:**
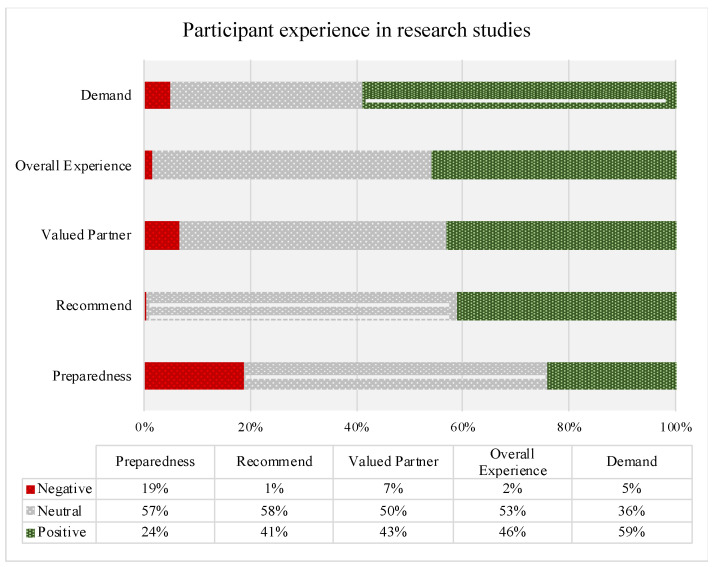
Overall Research Experience, Burden, and Preparedness.

Figure 8 illustrates the results from the Overall Research Experience, Burden, and Preparedness section. When participants reported prior research participation, many (46%) reported the highest positive rating (top box ratings of 9 or 10) about their experience in research, with 87.9% of responses being a 6 or higher. A significant portion (41%) of participants noted that they would “definitely” recommend that others participate in research. When asked about being a valued partner in the research process, 43% of respondents reported they always felt similar to a valued partner. Most participants (59%) reported a low level of burden of prior research participation. When asked about whether they felt prepared by the study team for their research experience, 24% of respondents reporting feeling completely prepared, and only 19% reported not feeling prepared. 

### 3.3. Research Priorities and Obstacles

**Figure 9:**
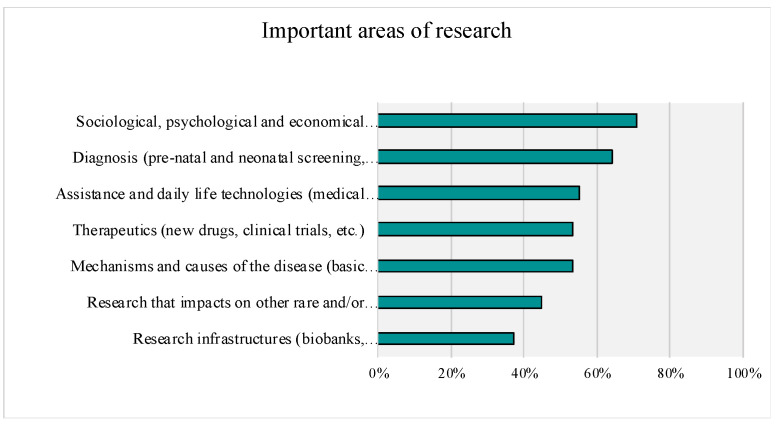
Important Areas of Research. Top-Box Answers are Presented (Rankings of 9 and 10).

When rating the importance of different types of research, using the top-box methods (ratings of 9 and 10), all but two areas (i.e., ‘research infrastructure’ with 38% top ratings, and ‘research that impacts other rare or common diseases’ with 46% of top ratings) received over 55% of top ratings (see Figure 9). The two areas that received the highest percentage of top ratings were psychosocial/quality of life research (70% top ratings) and diagnostic studies (66% top ratings). 

**Figure 10:**
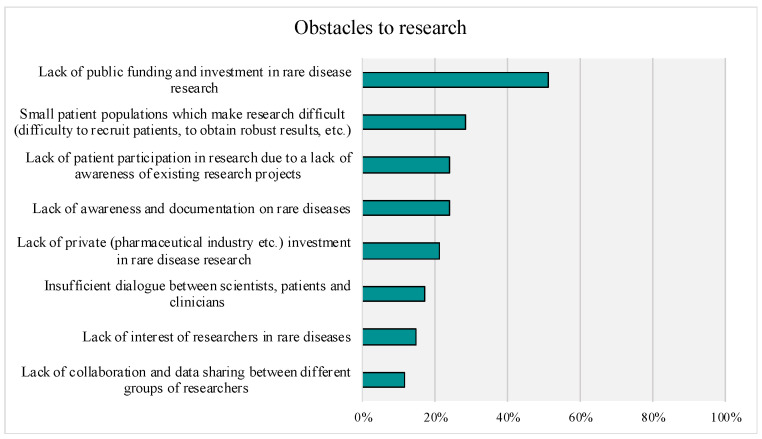
Obstacles to Research. Top-Box answers ( “Largest Obstacle” rankings) are Presented.

For research obstacles, the most often ranked “first largest obstacle” was lack of public funding, with 51% of participants ranking it as the top obstacle. The response option labeled as “other” also received 58% of “largest obstacle” ratings (see Figure 10). 

## 4. Discussion

The current study aimed to better understand global perspectives on research participation and research processes of individuals affected by a rare CNV and their families, spanning 29 countries and six languages. In this population at high risk for medical and neuropsychiatric outcomes, it is imperative that research teams strive to achieve a partnership with study participants to raise awareness and grow the field at large; ultimately leading to more meaningful scientific discovery. The findings complement prior work investigating patient-centered research participation [5,18,19], offer new insights into how rare CNV stakeholders view disease-focused research, and highlight several areas for improvement across research studies.

Our study highlights education and altruism as two primary top-rated motivators for participants joining and remaining in a research study. This is consistent with previous reports that have explored patient perspectives and factors prompting research participation [20,21,22]. Beyond education and altruism, more than half of the respondents gave a “highest importance” rating to gaining access to new therapies and treatments as a motivator to join and remain in a study. Relatedly, over 60% of respondents reported a top reason for staying enrolled in research is to improve health and/or quality of life. Relatedly, it was clear that stakeholders felt that shared results and progress updates within studies are very important for future research participation. Thus, it will be essential for future research to incorporate shared results into their protocols. Access to the care offered as part of research as well as laboratory and research results are also critical to the rare CNV community and should lead to discussions and improvement on the clinical care participants are receiving. It will also be key for the research team to be mindful of ethically presenting the research to avoid therapeutic and diagnostic misinformation [23].

In the current cohort, only 4.8 % of respondents reported that monetary compensation was a top reason for study participation. These findings were initially surprising to the committee, as it was hypothesized that reimbursement for time and travel would be a highly rated motivator for research participation. These are similar to findings in non-CNV groups where the participants’ altruism and a sense of connection to the research were the main drivers of participation, while financial compensation was not rated as a significant factor [18,19]. In sum, rare-CNV stakeholders’ main reasons for joining and remaining in research are true interests in helping others, learning more about their own rare CNV, and the potential to receive treatment and health improvements resulting from research protocols. 

Satisfaction with research participation is clearly an important component to understanding rates of current CNV stakeholder research participation and will likely foretell future participation. Our findings reveal that almost 90% of stakeholders in general report they were satisfied with their prior research participation, with 47% endorsing the highest-ranked level of positive experiences. The extant work examining research participation feedback in non-CNV cohorts finds that the majority of participants also rate their research experience as positive [18,19]. In one study aggregating feedback across a wide array of NIH-supported clinical centers, the relationship quality with the research team was the most significant contributor to satisfaction with their participation [19]. 

Prior work has shown that the informed consent processes are generally thorough and informative, but previous work demonstrates room for improvement in preparing participants for study activities [19]. In general, the current respondents reported they were prepared by the study team for the research procedures with less than 20% of respondents reporting that they did not feel prepared. Moreover, less than 10% of participants reported that they did not feel similar to valued research partners and only 5% reported a negative research burden (i.e., too high research demands). These stakeholders report largely positive outcomes and experiences with research. However, areas for improvement will include working with stakeholders on ways to better include their research questions, contribute to relevance and feasibility, and prepare all participants, so that they feel similar to valued members of the research process [24]. 

Little is known about Stakeholders’ views about the obstacles that investigative teams face today. Most respondents identified the lack of public funding for rare disease research as the greatest obstacle that future research faces. The remaining identified obstacles, such as small patient populations, lack of awareness, and lack of patient participation, are all related to the rarity of CNVs. Overall, respondents selected obstacles related to the research team less frequently, suggesting stakeholders view research hindrances as external rather than internal to study teams.

There were several limitations to the current study. First, the majority of respondents were mothers of children affected with a rare disease from the United States, despite there being representation from 29 countries: the pattern of results may be different in affected individuals compared to their caretakers and across a wider representation from different countries. Next, 22q11.2 deletion and duplication syndromes were the most common rare CNVs represented amongst the respondents. In addition to the relatively higher prevalence and knowledge base of these CNVs, this is likely a result of the positive relationship many of the investigators have with families, community members, and groups affected by 22q11.2 CNVs. Thus, creating better partnerships with the rare CNV community is likely a key area for improved research participation.

## 5. Conclusions

The current results provide a glimpse into how those affected with a rare CNV and their families feel toward research participation and their overall experience in rare disease research. It also reports on factors that motivate families to join, leave, and remain in research studies, and reveals stakeholders’ opinions on research priorities and obstacles to participation. Findings from this international survey can help advance future research, help investigative teams form better partnerships with the rare CNV community, and improve the experience of stakeholders’ and investigators alike. The G2MH hopes to address the issues raised in the current study about rare CNV research by incorporating participants’ feedback in research questions and study design and by pooling resources and continuing to analyze data across rare CNVs.

## Figures and Tables

**Table 1:** Participant Demographics.

Status and Relationship		Percentage of Respondents Reporting				Mean Age in Years (SD)	
	N	Female	United States	Urban or Suburban	Previous Participation	Respondent	Child
***Unaffected***							
Parent	575	85%	59%	79%	43%	44 (10)	13 (9)
Other Family Member	37	94%	73%	88%	10%	49 (17)	-
***Affected***							
Parent	33	82%	42%	61%	47%	43 (11)	11 (9)
Individual	27	68%	40%	86%	45%	33 (14)	-
***Declined to answer***							
	32	100%	33%	63%	44%	36 (14)	-
***Total***							
	**704**	**85%**	**51%**	**78%**	**41%**	**44 (11)**	**13 (9)**

**Table 2:** Represented Rare Copy Number Variants (CNVs).

Specific CNV Type	N	%
22q11.2 deletion	470	66.8
22q11.2 duplication	72	10.2
16p11.2 deletion	31	4.4
16p11.2 duplication	11	1.6
15q11.2 duplication	6	0.9
15q11.2 deletion	2	0.3
1q21.1 duplication	5	0.7
1q21.1 deletion	3	0.4
2p16.3 deletion	3	.04
2p16.3 duplication	1	0.1
17p11.2 deletion	2	0.3
17p11.2 duplication	1	0.1
15q13.3 duplication	3	0.4
7q11.23 deletion	3	0.4
Other	41	5.8
Declined to answer	50	7.1

## Data Availability

The data presented in this study are available on request from the corresponding author.

## Supplementary Materials

The following supporting information can be downloaded at: https://www.mdpi.com/article/10.3390/genes14010169/s1, Table S1: Rankings of reasons for joining a research study by region; Table S2: Rankings of reasons for staying enrolled in a research study by region; Table S3: Rankings of reasons for leaving a research study by region; Table S4: Important factors for future research by region; Table S5: Important areas of research by region; Table S6: Obstacles to research by region.
