# Supplementary material for: Gathering the Stakeholder’s Perspective: Experiences and Opportunities in Rare Genetic Disease Research
Source: Genes (Basel). 2023 Jan 7;14(1):169. doi: 10.3390/genes14010169 (PMC9859499; doi:10.3390/genes14010169)
Supplement: Supplementary file 1 [file genes-14-00169-s001.zip › genes-2043747-supplementary.pdf]

Region Analyses. To understand if survey responses differed by region, the 29 countries were collapsed into region groupings based on continents for statistical and interpretability purposes. Region results are only presented for Europe, North and South America, and Oceania; the Ns were too small for Africa (N=3 participants) and Asia (N= 4 participants). Chi-squared analyses were conducted to test for differences in survey responses by region.

For prior research participation, 56.7% of European respondents, 53.9% of North American respondents, 54.5% of South America respondents, and 63.6% of Oceania respondents reported never participating in prior research. Regions did not differ in the types of prior research studies that had previously participated (all *p*s > .06). When rating past experience with research, the percent endorsing the highest positive experience by region was: 21% of European respondents, 18.3% of North American respondents, 36.4% of South America respondents, and 9.1% of Oceania respondents, which was significantly different across regions $\mathcal{X}^2(6)=12.55$, $p=.05$. Regions also differed in their reports of feeling like a valued research partner ($\mathcal{X}^2(6)=14.93$, $p=.02$); the reporting of "always" feeling valued was: 26.8% of European respondents, 21.2% of North American respondents, 43.2% of South America respondents, and 21.2% of Oceania respondents. Regions did not differ on reports of study demands, preparedness for study by research team, or recommendation of research participation to others. The tables below list the additional survey items by region.

Supplement Table S1: Rankings of reasons for joining a research study by region. Top box responses (very important) are presented.

	**Joining Research**			
	**Europe (n=157)**	**North America (n=382)**	**South America (n=44)**	**Oceania (n=33)**
To find out more about my disease	79.6	80.1	95.5	81.8
Because no other medical options were available	21.0	26.2	29.5	15.2
**To gain access to new treatment/therapy***	**51.6**	**61.8**	**63.6**	**39.4**
To obtain free healthcare	19.1	18.1	27.3	18.2
To help others	66.2	70.2	70.5	72.7
Because of the Research/Health Center's reputation	19.1	28.8	31.8	21.2
**Because I am interested about the topic of study***	**52.2**	**51.6**	**77.3**	**51.5**
To obtain education and learning	53.5	58.6	75.0	48.5
**Because of a positive experience in another study***	**18.5**	**12.8**	**34.1**	**9.1**
**Because of family influence/involvement***	**33.1**	**23.8**	**40.9**	**18.2**
To earn money/payment	3.8	5.5	2.3	0.0
Because my caregiver encouraged me	4.5	6.8	6.8	9.1

*=significant (p<.05) Chi--squared differences across regions on this given item

Supplement Table S2: Rankings of reasons for staying enrolled in a research study by region. Top box responses (very important) are presented.

	**Staying Enrolled Research**			
	**Europe (n=68)**	**North America (n=180)**	**South America (n=23)**	**Oceania (n=13)**
To find out more about my disease	39.5	41.1	50.0	33.3
Because no other medical options were available	15.3	13.6	22.7	9.1
To gain access to new treatment/therapy	25.5	28.8	31.8	18.2
To obtain free healthcare	4.5	3.9	13.6	6.1
To help others	32.5	35.9	50.0	33.3
Because of the Research/Health Center's reputation	12.1	15.7	11.4	12.1
Because I am interested about the topic of study	26.8	28.3	45.5	24.2
**To obtain education and learning***	**19.7**	**28.3**	**45.5**	**21.2**
**Because of a positive experience in another study***	**15.3**	**9.4**	**27.3**	**9.1**
**Because of family influence/involvement***	**13.4**	**8.1**	**18.2**	**0.0**
To earn money/payment	1.9	2.1	0.0	0.0
Because my caregiver encouraged me	3.2	3.7	4.5	6.1
Because of my relationship with the research team	10.2	11.0	15.9	3.0
Feeling valued as a research participant	15.9	20.7	20.5	15.2
Improved health or quality of life	26.8	30.9	38.6	18.2

*=significant (*p*<.05) Chi-squared differences across regions on this given item

Supplement Table S3: Rankings of reasons for leaving a research study by region. Top box responses (very important) are presented.

	**Percent Endorsed of "Very Important"**			
	**Joining Research**			
	**Europe (n=68)**	**North America (n=180)**	**South America (n=23)**	**Oceania (n=13)**
Pain or discomfort related to participation	13.4	20.9	15.9	9.1
Worried about risks of treatment	18.5	24.3	22.7	18.2
Side effects that occurred during the study	19.7	23.3	29.5	15.2
Invasion of privacy	6.4	7.1	4.5	9.1
Too much time spent waiting around	3.8	7.3	0.0	0.0
**Time commitment required***	**4.5**	**10.2**	**0.0**	**6.1**
Family/work issues unrelated to the study	5.1	8.4	4.5	0.0
Interactions with research team	5.1	7.6	15.9	9.1
Not getting test results	10.8	15.7	27.3	12.1
**Undue pressure to stay in study***	**5.0**	**6.0**	**22.7**	**6.1**
**Problems with study payments***	**2.5**	**1.6**	**11.4**	**0.0**
**Unexpected tests and procedures that occurred during the study***	**3.8**	**7.9**	**20.5**	**9.1**
Transportation/parking	5.7	6.8	0.0	0.0

*=significant (*p*<.05) Chi-square differences across regions on this given item

Supplemental Table S4: Important factors for future research by region. Top-box responses are presented.

	**Percent Top Endorsed Reason**			
	**Europe (n=157)**	**North America (n=382)**	**South America (n=44)**	**Oceania (n=33)**
Access to computer, internet, and television	34.4	35.6	15.9	33.3
**Payment/More Payment***	**8.9**	**20.2**	**9.1**	**6.1**
**Support groups***	**36.3**	**30.4**	**52.3**	**36.4**
Volunteer appreciation	15.3	15.4	13.6	24.2
Flexible Schedule	47.8	56.5	45.5	54.5
Accessible parking and study location	28.9	34.0	20.5	21.2
Planned discharge and proper goodbye to research team	17.8	13.1	20.5	27.3
Summary of overall research results shared with me	76.6	79.1	72.7	75.8
**Results of personal lab tests shared with me or my doctor***	**68.8**	**77.7**	**63.6**	**63.6**

*=significant (*p*<.05) Chi-square differences across regions on this given item

Supplemental Table S5: Important areas of research by region. Top-Box answers are presented (rankings of 9 and 10)

	Percent Ranked First			
	**Europe (n=157)**	**North America (n=382)**	**South America (n=44)**	**Oceania (n=33)**
Mechanisms and causes of the disease (basic research)	53.5	50.3	59.1	48.5
Diagnosis (pre-natal and neonatal screening, gene testing, imaging, biological markers, etc.)	54.1	59.9	56.8	66.7
Therapeutics (new drugs, clinical trials, etc.)	49.7	50.3	50.0	39.4
Assistance and daily life technologies (medical devices, telemedicine, wheelchair, etc.)				
**Sociological, psychological and economical aspects of disease (quality of life assessment and social science)***	**58.6**	**64.1**	**72.7**	**45.5**
**Research infrastructures (biobanks, bioinformatics, etc.)***	**38.2**	**30.9**	**63.6**	**24.2**
**Research that impacts on other rare and/or common diseases***	**40.8**	**40.8**	**56.8**	**40.8**

*=significant (*p*<.05) Chi-square differences across regions on this given item

Supplemental Table S6: Obstacles to research by region. Top-Box answers (first largest obstacle) are presented

	Percent Endorsed First Largest Obstacle			
	**Europe (n=103)**	**North America (n=242)**	**South America (n=28)**	**Oceania (n=22)**
Small patient populations which make research difficult (difficulty to recruit patients, to obtain robust results, etc.)	10.8	14.1	11.4	12.1
Lack of public funding and investment in rare disease research	38.2	29.6	38.6	33.3
Lack of interest of researchers in rare diseases	3.2	6.5	9.1	6.1
Lack of private (pharmaceutical industry etc.) investment in rare disease research	8.9	4.5	11.4	6.1
Lack of awareness and documentation on rare diseases	12.1	12.6	11.4	9.1
Lack of patient participation in research due to a lack of awareness of existing research projects	8.3	15.2	4.5	18.2
Insufficient dialogue between scientists, patients, and clinicians	11.5	7.9	4.5	39.1
Lack of collaboration and data sharing between different groups of researchers	3.2	3.1	4.5	0.0

No region difference emerged on any of the above listed obstacles
